# Non-therapeutic laparotomies in military trauma (2009–2014)

**DOI:** 10.1007/s00464-024-11102-4

**Published:** 2024-08-14

**Authors:** Patrick F. Walker, Joseph D. Bozzay, David W. Schechtman, Faraz Shaikh, Laveta Stewart, M. Leigh Carson, David R. Tribble, Carlos J. Rodriguez, Matthew J. Bradley

**Affiliations:** 1 Walter Reed National Military Medical Center, 8901 Wisconsin Ave, Bethesda, MD 20814 USA; 2 Brooke Army Medical Center, JBSA Fort Sam Houston, TX USA; 3 Infectious Disease Clinical Research Program, Department of Preventive Medicine and Biostatistics, Uniformed Services University of the Health Sciences, Bethesda, MD USA; 4 Henry M. Jackson Foundation for the Advancement of Military Medicine, Inc, Bethesda, MD USA; 5 Texas Health Resources, Fort Worth, TX USA

**Keywords:** Non-therapeutic laparotomy, Exploratory laparotomy, Combat trauma, Military, Surgical site infections

## Abstract

**Background:**

Combat casualties are frequently injured in austere settings where modern imaging modalities are unavailable. Exploratory laparotomies are often performed in these settings when there is suspicion for intra-abdominal injury. Prior studies of combat casualties reported non-therapeutic laparotomy (NTL) rates as high as 32%. Given improvements in combat casualty care over time, we evaluated NTLs performed during later years of the wars in Iraq and Afghanistan.

**Methods:**

Military personnel with combat-related injuries (6/1/2009–12/31/2014) who underwent exploratory laparotomy based on concern for abdominal injury (i.e. not performed for proximal vascular control or fecal diversion) and were evacuated to Landstuhl Regional Medical Center (Germany) before being transferred to participating U.S. military hospitals were assessed. An NTL was defined as a negative laparotomy without substantial intra-abdominal injuries requiring repair. Characteristics, indications for laparotomy, operative findings, and outcomes were examined.

**Results:**

Among 244 patients who underwent laparotomies, 41 (16.8%) had NTLs and 203 (83.2%) had therapeutic laparotomies (i.e. positive findings). Patients with NTLs had more computed tomography scans concerning for injury (48.8% vs 27.1%; *p* = 0.006), less penetrating injury mechanisms (43.9% vs 71.9%; *p* < 0.001), and lower Injury Severity Scores (26 vs 33; *p* = 0.003) compared to patients with therapeutic laparotomies. Patients with NTLs were also less likely to be admitted to the intensive care unit (70.7 vs 89.2% for patients with therapeutic laparotomies; *p* = 0.007). No patients with NTLs developed abdominal surgical site infections (SSI) compared to 16.7% of patients with therapeutic laparotomies (*p* = 0.002). There was no significant difference in mortality between the groups (*p* = 0.198).

**Conclusions:**

Our proportion of NTLs was lower than reported from earlier years during the wars in Iraq and Afghanistan. No infectious complications from NTLs (i.e. abdominal SSIs) were identified. Nevertheless, surgeons should continue to have a low threshold for exploratory laparotomy in military patients in austere settings with concern for intra-abdominal injury.

In combat settings, penetrating trauma from explosive blasts and high-velocity gunshot wounds can often result in severe intra-abdominal injuries. Rapid diagnosis and management of abdominal trauma are crucial as delays can result in significant morbidity and mortality. Modern imaging modalities, such as computed tomography (CT) scans, are frequently unavailable in austere combat environments. Adjuncts, such as ultrasound or diagnostic peritoneal aspiration, can be helpful, but may result in equivocal findings. As a result, surgeons must often rely solely on physical examination and their judgment when deciding whether to perform an exploratory laparotomy. While this approach can be lifesaving, it also risks conducting non-therapeutic surgeries, which may result in the development of complications, such as surgical site infections (SSI), during a patient’s course of care.

The high frequency of blast trauma during the wars in Iraq and Afghanistan led to the use of exploratory laparotomies to evaluate for intra-abdominal injuries. After nearly a decade of combat, the occurrence of non-therapeutic laparotomies (NTLs) was assessed among U.S. and U.K. combat casualties [1, 2]. From 2002 to 2011, 32% of 1273 laparotomies performed among wounded U.S. military personnel were classified as NTL with a higher proportion occurring among casualties wounded in Afghanistan (38.2% vs 28.6% in Iraq) and peaking from 2007 to 2010 [1]. Among 130 wounded U.K. military personnel (2003–2011) who underwent a laparotomy, 20.8% were NTLs [2]. While the number of overall laparotomies increased over the study years, the proportion of NTLs showed a downward trend [2]. Given the early trends of NTLs, advances in combat casualty care and increased availability of resources (i.e. CT scan) as the military trauma system matured, we sought to examine the occurrence and characteristics of NTLs from the later years of the conflicts in Iraq and Afghanistan (2009–2014). The results could help inform guidelines and areas for performance improvements with combat casualty care.

## Methods

This retrospective observational study examined data from combat casualties collected as part of the Trauma Infectious Disease Outcomes Study [3, 4]. Inclusion in TIDOS required being active-duty military personnel ≥ 18 years of age who sustained injuries while deployed between 1 June 2009 and 31 December 2014 and were medically evacuated to Landstuhl Regional Medical Center (LRMC) in Germany before being transitioned along the continuum of care to a participating military hospital in the United States. The hospitals in the United States were Walter Reed National Military Medical Center (Walter Reed Army Medical Center and National Naval Medical Center prior to September 2011) and Brooke Army Medical Center. For this analysis, a further criterion was undergoing an exploratory laparotomy based on suspicion of intra-abdominal injury without diagnostic certainty (i.e. laparotomy was not performed for proximal vascular control of junctional or extremity hemorrhage or for fecal diversion). For individuals who had multiple laparotomies, data from the index laparotomy were utilized. This study was approved by the Uniformed Services University of the Health Sciences Institutional Review Board.

Patient demographics, injury characteristics (i.e. Injury Severity Score [ISS] and mechanism of injury), and early trauma care data (i.e. blood transfusion requirements) were obtained from the Department of Defense Trauma Registry (DoDTR) [3, 5]. Infection-related information (i.e. SSIs) was collected via the DoDTR TIDOS Infectious Disease module [6]. Data related to the indications for the exploratory laparotomy and resulting operative findings for therapeutic laparotomies (defined as having positive findings necessitating repair, resection, ligation, or other intra-abdominal intervention, including traumatic fascial defects large enough to require repair) were retrospectively abstracted from medical records. For this study, NTL was defined as a negative laparotomy without identification of significant intra-abdominal injuries requiring repair.

Characteristics, indications for laparotomies, and outcomes were compared between patients who had an NTL and those with therapeutic laparotomies. Statistical analysis was performed using chi-squared or Fisher’s exact tests for categorical variables, and the Kruskal–Wallis test for continuous variables. A *p*-value < 0.05 was deemed statistically significant. Statistical analysis was performed using SAS version 9.4 (Cary, NC).

## Results

### Study population

Among 341 combat casualties who underwent an exploratory laparotomy, 80 patients were excluded based on fecal diversion, proximal vascular control, or planned reoperation as the reason for the laparotomy. An additional 17 patients did not meet criteria (e.g. indication for negative laparotomy unknown and negative laparotomy performed after admission to LRMC) and were excluded. Among the 244 (71.6%) patients with laparotomies who met criteria for inclusion in the analysis, 41 (16.8%) patients had a laparotomy classified as NTL, while 203 (83.2%) patients had a therapeutic laparotomy. The patients were predominantly young (median age of 24 years) males (98.4%) who sustained injuries in Afghanistan (94.3%) while on foot patrol (70.1%; Table 1). Patients with an NTL had a higher proportion of blast-related injuries (82.9%; 65.9% injured by improvised explosive device [IED]) compared to those with a therapeutic laparotomy (61.6%, 43.4% injured by IED; *p* < 0.001). The level of care for the NTL was 44% occurring at a non-hospital medical unit within the combat zone and 56% occurring at a combat support hospital compared to 49% and 50%, respectively, for those who had a therapeutic laparotomy (*p* = 0.729). The NTL group had lower blood transfusion requirements within the 1st 24 h of injury (21.9% requiring ≥ 10 units vs 44.3%; *p* = 0.002), and while the majority were admitted to the intensive care unit (ICU, 70.7%), the proportion was less than that of those who had therapeutic laparotomies (89.2%; *p* = 0.007). The overall injury burden for the entire population was primarily classified as critical injury severity (i.e. ISS ≥ 26), including patients with NTLs (73.8% with ISS ≥ 26). Nevertheless, patients with NTLs did have lower injury severity compared to patients with therapeutic laparotomies (median ISS of 26 vs 33; *p* = 0.003).

**Table 1:** Characteristics of combat casualties who underwent exploratory laparotomies with diagnostic uncertainty

Characteristic, No. (%)	Total Patients (*n* = 244)	Patients with NTL (*n* = 41)	Patients with TL (*n* = 203)	*p*-value
Male	240 (98.4)	40 (97.6)	200 (98.5)	0.523
Age, median years (IQR)	24 (21–28)	24 (22–30)	24 (21–28)	0.187
Mechanism of Injury				
Blast	159 (65.2)	34 (82.9)	125 (61.6)	< 0.001
IED Blast	115 (47.1)	27 (65.9)	88 (43.4)	
Non-IED Blast	44 (18.0)	7 (17.1)	37 (18.2)	
Non-Blast	85 (34.8)	7 (17.1)	78 (38.4)	
Injured on foot patrol	171 (70.1)	26 (63.4)	145 (71.4)	0.307
Injured in Afghanistan	230 (94.3)	38 (92.7)	192 (94.6)	0.711
Level of care for exploratory laparotomy^a^				0.729
Non-hospital medical unit in combat zone (Role 2)	117 (47.9)	18 (43.9)	99 (48.8)	
Combat support hospital (Role 3)	124 (50.8)	23 (56.1)	101 (49.7)	
LRMC	2 (1.0)	0	2 (1.0)	
Injury Severity Score				
Median (IQR)	31 (24–43)	26 (16–34)	33 (26–43)	0.003
0–9 (minor)	10 (4.1)	7 (17.1)	3 (1.5)	< 0.001
10–15 (moderate)	9 (3.7)	2 (4.9)	7 (3.5)	
16–25 (severe)	45 (18.4)	10 (24.4)	35 (17.2)	
≥ 26 (critical)	180 (73.8)	22 (53.7)	158 (77.8)	
Shock index, median (IQR)	0.9 (0.7–1.3)	0.8 (0.6–1.0)	0.9 (0.7–1.3)	0.105
Blood units transfused in 1st 24 h post-injury				
Median (IQR)	10 (4–20)	7 (5–17)	11 (4–22)	0.507
0 or missing	57 (23.4)	19 (46.3)	38 (18.7)	0.002
1–9	88 (36.1)	13 (31.7)	75 (37.0)	
10–20	52 (21.3)	6 (14.6)	46 (22.7)	
> 20	47 (19.3)	3 (7.3)	44 (21.7)	
Indications for laparotomy				
Penetrating abdominal injury	164 (67.2)	18 (43.9)	146 (71.9)	< 0.001
Hypotension	79 (32.4)	7 (17.1)	72 (35.5)	0.022
Positive FAST	32 (13.1)	5 (12.2)	27 (13.3)	0.848
Positive CT	75 (30.7)	20 (48.8)	55 (27.1)	0.006
ICU admission				0.007
None	34 (13.9)	12 (29.3)	22 (10.8)	
LRMC only	43 (17.6)	7 (17.1)	36 (17.7)	
U.S. military hospital ± LRMC	167 (68.4)	22 (53.7)	145 (71.4)	
Hospitalization LOS, median days (IQR)	28.5 (16–47.5)	26 (12–35)	30 (16–52)	0.044

*CT* computed tomography, *FAST* focused assessment sonograph trauma, *ICU* intensive care unit, *IED* improvised explosive device, *IQR* interquartile range, *LRMC* Landstuhl Regional Medical Center, *NTL* non-therapeutic laparotomy, *TL* therapeutic laparotomy

^a^One patient with a therapeutic laparotomy had it occur prior to arrival at LRMC; however, whether it was at Role 2 or Role 3 is missing

### Laparotomy characteristics and outcomes

Among the 244 patients explored, 67.2% had their laparotomy performed for a penetrating abdominal injury with suspicion of a transperitoneal trajectory (Table 1). There were 32.4% of patients who presented with hypotension prior to undergoing surgery. Only 13.1% of patients had a positive focused assessment with sonography for trauma (FAST), and 30.7% of patients had a positive pre-operative CT scan.

Patients with NTLs had more positive pre-operative CT scans concerning for injury (48.8% vs 27.1%, *p* = 0.006) compared to patients with therapeutic laparotomies. In addition, the patients with NTLs had a lower proportion of hypotension (17.1% vs 35.5%; *p* = 0.022) and penetrating injuries (43.9% vs 71.9%; *p* < 0.001) as the indication for the laparotomy. There was no statistical difference in occurrence of positive FAST scans between the groups.

Among the 203 patients with therapeutic laparotomies, the most common injured organs were colon (33%), small bowel (31%), spleen (24.6%), and liver (20.2%; Table 2). Additional injured organs included kidney (11.8%), bladder (9.9%), rectum (10.8%), stomach (4.9%), diaphragm (10.3%), pancreas (3.5%), duodenum (3.0%), and extrahepatic bile ducts (2.0%). Although 25.1% of patients had a retroperitoneal hematoma noted, only 7.9% had a major abdominal vascular injury. This included 5.9% of patients with injuries to the iliac vessels, 2% with inferior vena cava injuries, and one patient (0.5%) with an injury to the inferior mesenteric artery.

**Table 2:** Injuries identified in therapeutic exploratory laparotomies

Injuries, No. (%)	Patients with TL (*n* = 203)
Colon	67 (33.0)
Small bowel	63 (31.0)
Retroperitoneal hematoma	51 (25.1)
Spleen	50 (24.6)
Liver	41 (20.2)
Kidney	24 (11.8)
Rectum	22 (10.8)
Diaphragm	21 (10.3)
Bladder	20 (9.9)
Major abdominal vascular injury	16 (7.9)
Inferior vena cava	4 (2.0)
Inferior mesenteric artery	1 (0.5)
Iliac artery/vein	12 (5.9)
Stomach	10 (4.9)
Pancreas	7 (3.5)
Duodenum	6 (3.0)
Biliary	4 (2.0)

*TL* therapeutic laparotomy

The occurrence of traumatic brain injury was similarly distributed across both groups, with an overall proportion of 47.1% (Table 3). Deep venous thrombosis and pulmonary embolism were observed in 14.6% and 4.9% of patients with NTLs, respectively, compared to 10.3% and 6.9% in the therapeutic laparotomy group, with no statistical significance. No patients in the NTL group had a post-operative open abdomen compared to 79.3% of patients in the therapeutic laparotomy group (*p* < 0.001). There were no instances of abdominal SSIs in the NTL group, whereas 16.7% of patients with therapeutic laparotomies developed an abdominal SSI (*p* = 0.002). One individual in the NTL group underwent a second laparotomy (also non-therapeutic), while patients in the therapeutic laparotomy group had a median of 3 laparotomies (interquartile range: 2–4). Patients with NTLs had a shorter duration of hospitalization compared to patients with therapeutic laparotomies (median of 26 vs 30 days; *p* = 0.044). There was no significant difference in the proportion of in-hospital mortality between the groups with the overall crude mortality being 2.1%.

**Table 3:** Outcomes of patients stratified by laparotomy classification

Outcomes, No. (%)	Total Patients (*n* = 244)	Patients with NTL (*n* = 41)	Patients with TL (*n* = 203)	*p*-value
Traumatic brain injury	115 (47.1)	21 (51.2)	94 (46.3)	0.565
Deep vein thrombosis	27 (11.1)	6 (14.6)	21 (10.3)	0.424
Pulmonary embolism	16 (6.6)	2 (4.9)	14 (6.9)	1.00
Post-operative open abdomen	161 (66.0)	0	161 (79.3)	< 0.001
Surgical site infection	34 (13.9)	0	34 (16.7)	0.002
Hospitalization LOS, median days (IQR)	28.5 (16–47.5)	26 (12–35)	30 (16–52)	0.044
Death	5 (2.1)	2 (4.9)	3 (1.5)	0.198

*IQR* interquartile range, *LOS* length of stay, *NTL* non-therapeutic laparotomy, *TL* therapeutic laparotomy

## Discussion

The findings of this study offer important perspectives on the frequency and outcomes of NTLs in combat casualties during the later years of the wars in Iraq and Afghanistan. The 16.8% proportion of NTLs identified in our population is notably lower compared to previously reported data. For instance, Morrison et al. [2] reported 20.8% of 130 U.K. military personnel who underwent laparotomies from 2003 to 2011 had NTLs. Similarly, Mitchell et al. [1] identified an NTL rate of 32.8% for U.S. military personnel wounded in Afghanistan and 28.6% for those injured in Iraq between 2002 and 2011, while Hathaway et al. [7] reported that 35% of combat casualties who had laparotomies conducted for indications other than proximal control (2009–2011) had negative findings. Our current research, demonstrating a lower proportion of NTLs, is indicative of the iterative advancements in combat casualty care, growing experience of the deployed surgeons, countermeasures to include improved body armor, and maturation of the military trauma system with increases in available resources [8]. Furthermore, it mirrors the evolution and refinement of Joint Trauma System clinical practice guidelines as more evidence becomes available.

Military trauma research generally exhibits higher rates of NTLs compared to civilian trauma settings. For instance, Haan et al. [9] reported 6% of 820 patients (1998–2001) had an NTL in a contemporary urban level 1 trauma center in the United States. In another U.S. civilian study, 3.9% of 1871 laparotomies conducted between 2003 and 2008 were identified as NTL [10]. The occurrence of NTLs at civilian centers may be affected by use of routine laparotomies related to injury mechanisms (e.g. generally higher rate of NTLs with abdominal stab wounds versus abdominal gunshot wounds) [11]. While a study of trauma patients in Denmark reported a higher proportion of NTLs (17.3%), it was likely the result of an institutional protocol requiring routine laparotomies when there was evidence of peritoneal penetration with stab wounds (77% of the patients with NTLs sustained stab wounds) [12]. Overall, our finding of a 16.8% proportion of NTLs aligns with expectations within the military context as being elevated in comparison to civilian data, which is not unexpected given the austere, resource-limited settings in which surgeons often operate. Complicating the military environment is the gap in surgical care during evacuation of the casualty to the next level of care.

Our study identified differences in specific pre-operative clinical and radiographic indicators between the patients who had an NTL versus a therapeutic laparotomy. In particular, nearly 50% of patients with NTLs had a false-positive CT scan indicative of a transperitoneal trajectory with no injuries requiring intervention identified upon exploration, in contrast to 27% among patients with therapeutic laparotomies. This calls into question whether metallic fragmentation from the higher proportion of IED blasts among the patients with NTLs (66% vs 43%) might compromise the precise determination of peritoneal violation via CT scanning. Unlike civilian trauma, where substantial data on the accuracy of CT scans in both blunt and penetrating abdominal trauma is available [13–15], the efficacy of CT scans in military abdominal trauma is still being explored. In one study, 29.4% of 60 combat casualties who underwent CT and subsequently required a laparotomy had an NTL [16]. In contrast, an NTL rate of 3.9% was reported among a group of 159 combat casualties with abdominal injuries who underwent CT scan [17]. A recent meta-analysis found that CT scans provided excellent diagnostic accuracy with military abdominal trauma; however, the authors noted the limited number of studies available for inclusion in the meta-analysis and that the types of abdominal injuries were not always well-described in the studies [18]. Selective use of non-operative management is restricted in military scenarios due to extended transport times and scarce resources for monitoring, as well as the potential lethal consequences for missed intra-abdominal injuries. Thus, further analysis of CT results with greater detail is warranted to identify the limitations of CT scans and develop best practices for their use in combat settings, particularly given advancements in CT technology.

The notable absence of SSIs in the NTL group in our study is interesting given the cases were likely not the most sterile procedures. Analysis of combat-related laparotomies conducted for indications other than proximal control reported 4% with wound infections and 3% with intra-abdominal abscesses; however, only 35% of the laparotomies were classified as non-therapeutic [7]. However, similar to our findings, a prior examination of U.S. military personnel with NTLs reported one intra-abdominal abscess and zero wound infections [1]. It should be noted that no patients with an NTL in our study were left with a post-operative open abdomen, which may have contributed to the lack of SSIs. Moreover, while a high proportion (71%) of patients in the NTL group were admitted to the ICU in our study, the need for ICU support was less than what was required for patients with therapeutic laparotomies. Although patients in the NTL group frequently had critical injuries, the reduced ICU requirement could potentially be attributed to their slightly lesser severity of injuries. Nevertheless, the data suggest limited short-term complications from NTL procedures; however, limited long-term follow-up impedes the evaluation of enduring complications. Considering the up to 19.1% incidence of incisional hernia post-laparotomy in combat casualties [19], continued monitoring is warranted. In that regard, laparoscopic capabilities were not available at many locations. However, the use of laparoscopy to explore the abdomen could have reduced the incidence of NTL and lowered the incisional hernia rate, especially in the setting of such high false-positive CTs.

Our analysis was constrained by its retrospective design, and inclusion criteria necessitated patient survival until repatriation to the United States. Documentation inconsistencies or absences for some patients, owing to their transfer through varying levels of care, also imposed a limitation. Lastly, comprehensive follow-up data for some patients were unattainable.

Despite the reduced proportion reported in our study, NTLs will persist in combat settings, especially with large-scale combat operations and prolonged-field care scenarios. The clinical presentation of combat patients requiring exploratory laparotomy for abdominal trauma can be obfuscated by multiple factors, such as concurrent injuries, the urgent need for decision-making, and potentially ambiguous pre-operative evaluations. Advancing our understanding through further research is important to refine triage criteria and pinpoint low-risk patients suitable for observation over surgery. Yet, it must be emphasized that in uncertain cases, exploratory laparotomy remains an indispensable intervention in optimizing survival in deployed environments.
